# Adolescents’ academic achievement and life satisfaction: the role of parents’ education

**DOI:** 10.3389/fpsyg.2015.00052

**Published:** 2015-02-03

**Authors:** Julia Crede, Linda Wirthwein, Nele McElvany, Ricarda Steinmayr

**Affiliations:** ^1^Department of Psychology, Technical University Dortmund, DortmundGermany; ^2^Institute for School Development Research (IFS), Technical University Dortmund, DortmundGermany

**Keywords:** academic achievement, life satisfaction, parent’s education, socio-economic status, social mobility

## Abstract

Drawing on the background of positive psychology, there has only recently been a focus on adolescents’ life satisfaction (LS) in the context of education. Studies examining the relationship between adolescents’ academic achievement and LS have shown conflicting results and the reasons are not fully understood. The present study investigated the role of parents’ education as a potential moderator of the relationship between adolescents’ academic achievement and LS. A sample of German high school students (*N* = 411) reported parents’ educational attainment, as an indicator of family socio-economic status, and students’ academic achievement was operationalized by grade point average in five subjects. Results indicated that only mothers’ education functioned as a moderator of the relationship between academic achievement and students’ LS. The association between academic achievement and LS was only found in the group of students whose mothers had achieved the same or a higher education (at least high school diploma) as their own children. Fathers’ educational attainment, however, was not a significant moderator of the respective relationship. Directions for future research and the differential influences of fathers’ and mothers’ education are discussed with regard to potential underlying processes.

## INTRODUCTION

In contrast to the common focus of many psychologists on psychopathology, within the framework of positive psychology the focus is on understanding the conditions that improve individuals’ life satisfaction (LS) (Seligman and Csikszentmihalyi, 2000; Suldo and Huebner, 2004). This focus has the potential to fundamentally enrich the study of psychological ill-being, as it not only includes individuals who are already at risk and show significant levels of psychopathological symptoms, but also healthy and normal-functioning individuals. Therefore, it contributes to the development of prevention and intervention programs (Greenspoon and Saklofske, 2001; Suldo and Shaffer, 2008; Stewart and Suldo, 2011).

In the context of positive psychology, many researchers have underlined the construct of LS, which deals with the elements that may characterize a ‘good life’ and is concerned with how individuals’ lives can be improved (Bullinger, 2009). Whereas studies investigating determinants of LS have previously focused on adulthood (Diener et al., 1999), there has recently been an increase in studies concentrating on children and adolescents (Huebner, 2004; Gilman and Huebner, 2006; Proctor et al., 2010).

As adolescence is a period characterized by emotional upheaval (Gilman and Huebner, 2003) as well as both exposure to more opportunities and risks (Chow, 2005), adolescents are at risk of developing psychological ill-being. Furthermore, they face a great number of challenges and stressors that are related to school. For this reason, research on adolescents’ LS has been based in the context of education, in both regular (Huebner, 1997) and in special education (Crocker, 2000). This reflects a significant shift in perspective away from a functionalist view on children and adolescents to the point of seeing them as more than just learners and promoting their rights and needs (Bullinger, 2009).

Because students face increased pressure to succeed academically today, especially in highly selective and academically rigorous schools, researchers have examined the relationship between students’ academic achievement and LS, and so far, studies focusing on this relationship have shown conflicting results. Whereas earlier research did not consistently find correlations, more recent studies have reported a positive relationship between adolescents’ academic achievement and LS (e.g., Kirkcaldy et al., 2004; Suldo et al., 2008). Therefore, researchers have emphasized the importance of examining any underlying mechanisms and potential moderating variables of this relationship (Suldo et al., 2006). As earlier studies on students’ LS and academic achievement have been criticized for not taking into consideration any demographic or contextual information (Suldo et al., 2006), studies concentrating on potentially moderating variables of this kind such as school environments or socio-economic background, are currently lacking. To address this research gap, the present study set out to examine whether parents’ education, as an indicator of students’ socio-economic status (SES), moderated the relationship between students’ academic achievement and LS.

### LIFE SATISFACTION IN ADOLESCENCE AND ACADEMIC ACHIEVEMENT

Life satisfaction is defined as the way an individual evaluates his or her past or current conditions of life (Myers and Diener, 1995; Huebner et al., 2005). LS judgments refer to either global statements or describe a person’s satisfaction with important domains of life, such as friends, family, or self (Suldo et al., 2006). With respect to youth, adolescents’ global LS reports are strongly correlated with ratings of satisfaction with family, friends, school, living environment, and self (Seligson et al., 2003).

The construct of LS is an aspect of the larger research area of subjective well-being, which is strongly influenced by the research of Diener and Lucas (1999). Here, LS represents one of three interrelated but distinct elements of subjective well-being, alongside the presence of positive affect, and the absence of negative affect (Diener, 2000; Huebner et al., 2004). Among these components of subjective well-being, LS is seen as the most stable as well as the key indicator of subjective well-being (Veenhoven, 1988; Suldo et al., 2006), which is why we chose to concentrate on LS in the present study.

During childhood and adolescence, academic achievement is important because in today’s society academic accomplishments as well as failures determine an individual’s future academic career and job opportunities (Kadison and DiGeronimo, 2004; Rana and Mahmood, 2010). There is a lot of theoretical support for the relationship between academic achievement and LS. Originally developed in the field of organizational psychology, the happy-productive worker hypothesis has frequently been adapted to an educational context. According to this thesis, the productivity of employees is closely linked to job satisfaction (Wright et al., 2002). Consequently, in the context of school, better-performing students may also demonstrate higher levels of LS.

To date, however, empirical studies examining the association between academic achievement and adolescents’ LS have revealed conflicting results. *Subjective* measures of academic performance such as self-evaluated or perceived academic competence have been shown to predict LS (Huebner et al., 1999; Suldo and Huebner, 2004, 2006). However, the relationship between *objective* indicators of academic achievement, i.e., achievement test scores or high school grade point average, and LS is less clear as results differ across age and different cultural and developmental groups (for an overview see Chang et al., 2003).

There have been some studies that have focused on the cross-sectional association between academic performance and LS on a national level. By using data from The Programme for International Students Assessment (PISA), Kirkcaldy et al. (2004) found strong correlations between the high school students of different nations’ average LS scores and achievement test scores (standardized achievement tests assessing scientific, mathematical, and reading literacy), the highest correlation to LS being reading achievement scores (*r* = 0.63). However, as Suldo et al. (2006) note, the analyses did not control for the countries’ differing economic and social factors. Furthermore, associations on a national level do not account for associations on an individual level.

Research on the association between academic achievement and LS that takes into consideration individual-level factors (e.g., family income, education level) is also unclear. In a number of cross-sectional studies, academic achievement was not related to students’ LS scores and no differences in levels of global LS between students with different achievement levels (e.g., students at risk for school failure and normally achieving students) were found (Huebner, 1991; Huebner and Alderman, 1993; Suldo et al., 2006). In contrast, other studies have suggested a moderate correlation (*r* = 0.32) between academic achievement and adolescents’ global LS in samples of *N* = 485 (Gilman and Huebner, 2006) and *N* = 349 adolescents (Suldo and Shaffer, 2008). Similarly, Verkuyten and Thijs (2002) showed that LS correlated significantly (*r* = 0.12) with the GPA averages in a sample of *N* = 1.090 Dutch students.

Longitudinal studies provide evidence that the correlation between academic performance and LS might be reciprocal, with evidence of an impact of LS on later school achievement indicated by grades and an effect of academic achievement on subsequent LS (Suldo et al., 2011). Consequently, these findings support a phenomenon which is called ‘the good circle,’ which suggests that high achievement scores may increase students’ LS which eventually motivates students to get better grades (Samdal et al., 1999).

### PARENTS’ EDUCATION AS A MODERATOR OF THE RELATIONSHIP BETWEEN ACADEMIC ACHIEVEMENT AND LIFE SATISFACTION

Whilst demographic variables contribute modestly to adolescents’ LS (Huebner et al., 2000; McCullough et al., 2000), the research has predominantly focused on intra-personal variables (for a review see Huebner et al., 2004). It is therefore important to examine contextual variables as potential moderators of academic achievement and LS in order to determine the conditions under which the magnitude of this relationship may become stronger or weaker.

Socio-economic status is one of the most widely used contextual variable in educational research (Sirin, 2005). It refers to an individual’s or a family’s ranking on society’s hierarchy with regard to access to valued commodities such as wealth, power, and/or social status (Mueller and Parcel, 1981; Sirin, 2005), and is often operationalized by parents’ income, education, and occupation as the three main indicators (Hauser, 1994; Sirin, 2005).

Studies examining the role of SES in the context of students’ LS have shown differing results. Many studies have not detected a relationship between students’ global LS and SES (Huebner, 1997; Konu et al., 2001). In contrast, other studies have found a positive relationship between SES and LS. Moreover, disadvantaged students from lower socio-economic backgrounds reported lower LS than higher SES students (e.g., Neto, 1993; Ash and Huebner, 2001). All studies did, however, only investigate a linear relationship between SES and LS, and we are not aware of any studies that have investigated whether SES is a moderator of the relationship between LS and academic success.

The idea that parents’ education as an indicator of family SES might be a potential moderator of the relationship between academic achievement and LS is supported by Bourdieu’ (1986) theory of class distinction. According to Bourdieu (1986), parents’ education strongly influences students’ academic success because more educated parents are able to provide more cultural and social capital that facilitates their children to succeed in school. Moreover, by providing their children with these forms of capital, more educated parents may better foster students’ motivation to succeed academically (Steinmayr et al., 2012) and have higher educational aspirations for their children (Zhan and Sherraden, 2003; Kim and Sherraden, 2011). This can affect two academic outcomes (cf. Steinmayr et al., 2014). First, academic success is indicated by high achievement in the school one attends. Second, in a tracked school system, educational success is linked to the type of school a student attends and the corresponding school leaving certificate.

Germany’s tracked school system is based on early ability streaming of students. In Germany, students are tracked into three different types of secondary schools at a relatively early point of their educational careers (mostly after fourth grade): the Hauptschule (the lowest level secondary school type), the Realschule (an intermediate level secondary school), or the Gymnasium (higher-level secondary school which enables students to pursue further academic studies; offering a Fachhochschulabschluss (students are only allowed to study at universities of applied sciences) or the Abitur (students are allowed to study at all universities; Mühlenweg, 2007). Furthermore, some federal states additionally offer comprehensive schools which give their students the possibility to obtain the school leaving certificate from the latter two school types (Realschule and Hauptschule) or from all three of them.

Expectations concerning their children’s success might be different for parents with a high and a lower education in a tracked school system. In a tracked school system, educational success is achieved if a student attends the highest school track. Well-educated parents who have attended the highest school track themselves often expect their children to attend the highest school track as they consider it the normal form of education compared to less educated parents who cannot underpin their judgment of high-tracked school with first-hand experience (Schnabel et al., 2002). Furthermore, well-educated parents are more willing to encourage their children to succeed at school out of fear of attaining a lower status symbolized by a lower school track (Goldthorpe, 2007). In contrast, children who attend a school track higher than the one their parents attended have already succeeded academically in their parents’ point of view. For them, their children’s actual school performance might not be as relevant because their children are already seen as more academically successful than themselves by attending a higher track in a tracked school system. In contrast, the encouragement by parents whose children attend the highest school track as they did can only refer to actual school performance in form of grades or achievement test results as their children cannot attend a school track higher than the one they attended themselves.

In light of the current societal changes, parents’ focus on their children’s education might become even stronger. As the educational level among the population in general has increased, families with higher SES consequently fear to encounter downward mobility, leading to a greater encouragement of their children’s education (Goldthorpe, 2007). If these children do not perform well in school the expectations of their parents are not met which might influence their children’s LS. As the discrepancy between an individual’s aspirations and actual standing is related to LS (Michalos, 1985), LS of students from high socio-economic backgrounds may be affected more strongly by their actual academic achievement than those of their peers from low socio-economic backgrounds if they attend the same school in a tracked school system and if students with low SES attend a higher school track than their parents did. Thus, for adolescents whose parents are less educated, students’ academic achievement may play a different role for their LS.

### HYPOTHESES

The present study aims at investigating the following hypotheses:

(1) Students’ academic achievement (GPA) is significantly positively correlated with LS scores.
(2) Parents’ education moderates the relationship between students’ academic achievement and LS. The association between students’ academic achievement and LS is lower for students who reached a higher level of educational attainment than their parents had, than for students who reached the same level of educational attainment as their parents.

## MATERIALS AND METHODS

### SAMPLE AND PROCEDURE

A total of 411 (49.1% female) 11th grade students (*M* = 16.42 years, *SD* = 0.54) participated in the present study. Students attended a type of German school that prepares them for university (“Gymnasium”), which is the most academic and prestigious secondary school track in Germany. Students were recruited from four different schools located in two mid-sized towns in Western Germany. Participation was optional and given during regular school day. Written consent was obtained from all parents of the under-aged students. The students were tested by trained research assistants and psychology university students. On the day of testing some students were ill, which resulted in an overall participation rate of about 92%. Students who were not considered did not significantly differ from this sample in any investigated variables. Our sample can be considered typical of the population from this type of school. The majority of students were Caucasian from medium to high SES homes. Students from disadvantaged and/or migration backgrounds are under-represented in this kind of school and it is attended by more girls than boys.

### MEASURES

#### Life satisfaction

Life satisfaction was measured using the General Life Satisfaction Scale developed by Dalbert (2003). It consists of seven items (e.g., “I am satisfied with my life.”, “I consider myself a happy person.”) measuring the cognitive dimension of subjective well-being. The scale describes satisfaction with one’s present and past life and future perspectives. The items of Dalbert’s General Life Satisfaction Scale are comparable with the items of the LS scale developed by Diener et al. (1985). In our sample Cronbach’s α coefficient was α = 0.89.

#### Academic achievement

The academic achievement measured was the GPA indicated on the students’ last report cards. The school delivered report cards for all students and therefore there was no missing data on this variable. In Germany, grades are coded on a six point scale where “1” indicates excellent achievement and “6” indicates the poorest achievement. Grades were reversed to facilitate interpretation of the results that higher scores were indicative of better performance. The subjects German and Math were mandatory for all students and thus all students had grades in these subjects. With regard to foreign language, science and social science, students were allowed to choose courses. Grades of the different courses within each category were summed up as indicators of academic achievement in these domains.

#### Parents’ education

We considered the educational level of both fathers and mothers. The information on parents’ education was gained from the students themselves, each indicating the highest educational degree their mother and father attained (cf. Steinmayr et al., 2010). The response options were: 0 = “No school leaving certificate at all,” 1 = “Hauptschulabschluss” (school leaving certificate from lowest school track), 2 = “Realschulabschluss” (school leaving certificate from middle school track), 3 = “Fachhochschulreife” (school leaving certificate from the highest school track that allows to study at universities of applied sciences, 4 = “Abitur” (school leaving certificate from the highest school track which allows to study at *all* universities), and 5 = “others”. None of the students used the category “others.” An overview of the classification of parents’ educational attainment in the present study can be seen in **Table 1**. The information about fathers’ educational level was distributed as follows: (0:*n* = 1; 1:*n* = 75; 2:*n* = 106; 3:*n* = 69; 4:*n* = 151). Mothers’ education was distributed as follows: (0:*n* = 0; 1:*n* = 73; 2:*n* = 46; 3:*n* = 166; 4:*n* = 118). Eight students did not report their mother’s school leaving certificate and ten students did not report their father’s school leaving certificate. Correlation between mothers’ and fathers’ educational level was *r_s_* = 0.13. As they were not strongly associated we considered our hypotheses separately for mothers and fathers.

**Table 1:** Classification of parents’ educational attainment in the present study.

Education track relative to child’s	English translation	Mothers’ educational attainment	Fathers’ educational attainment
Low	No school leaving certificate	0%	0.2%
Low	School leaving certificate from lowest school track	17.8%	18.2%
Low	School leaving certificate from middle school track	11.2%	25.8%
High	School leaving certificate from the highest school track. Allows study at universities of applied sciences	40.4%	16.8%
High	School leaving certificate from the highest school track. Allows study at *any* universities*	28.7%	36.7%
NA	Other	0%	0%

*The educational track of the students of this study.

### STATISTICAL ANALYSES

There were some missing values in the LS measure (0 < 1%) and we estimated missing values by applying the full information maximum likelihood estimator (FIML; Little and Rubin, 1987). We dichotomized the variables mother’s education and father’s education into two subgroups; low and high educational background, classified on the basis of the mothers’ and fathers’ highest level of educational attainment, respectively. If the mother had completed the same or a higher level of education than the child, that child was classified as having high educational background of the mother, and students whose mother had completed a lower level of education were classified as having low educational background of the mother. Father’s educational attainment was classified the same way. Children whose parents’ level of education was lower than their own (e.g., school leaving certificates of Realschule or Hauptschulabschluss) were compared to groups in which parents’ level of education was the same or higher as their children’s (e.g., Fachhochschule or Gymnasium). We checked for differential effects for mothers’ and fathers’ education, and although not all students reported both parents’ information, and the sample sizes thus slightly differed, we did not find any systematic differences between groups that reported the school leaving certificate of both parents or only of one parent.

To examine the postulated moderator effect, we used moderated regression analyses to predict LS. Before conducting interaction analyses, all predictors in the entire sample were centered (Aiken and West, 1991). The two predictors (academic achievement and mothers’ or fathers’ education) and the interaction term were included in the regression analyses for each model. The statistical significance of the interaction term provides evidence for the moderating effect (Aiken and West, 1991). To compute the moderator analyses we used the program Interaction (Soper, 2013).

## RESULTS

### DESCRIPTIVE STATISTICS

Descriptive statistics and inter-correlations of all measures are presented in **Table 2** and were similar to the coefficients found in the respective manual (Dalbert, 2003).

**Table 2:** Intercorrelations of students’ grades and life satisfaction (LS) measures.

	Descriptives			Intercorrelations
	*M*	*SD*	α	
(1) GPA	2.87	0.58		0.14**
(2) Life satisfaction	5.10	1.07	0.89	

**Correlation is significant at the 0.01 level (two-tailed).

**FIGURE 1:**
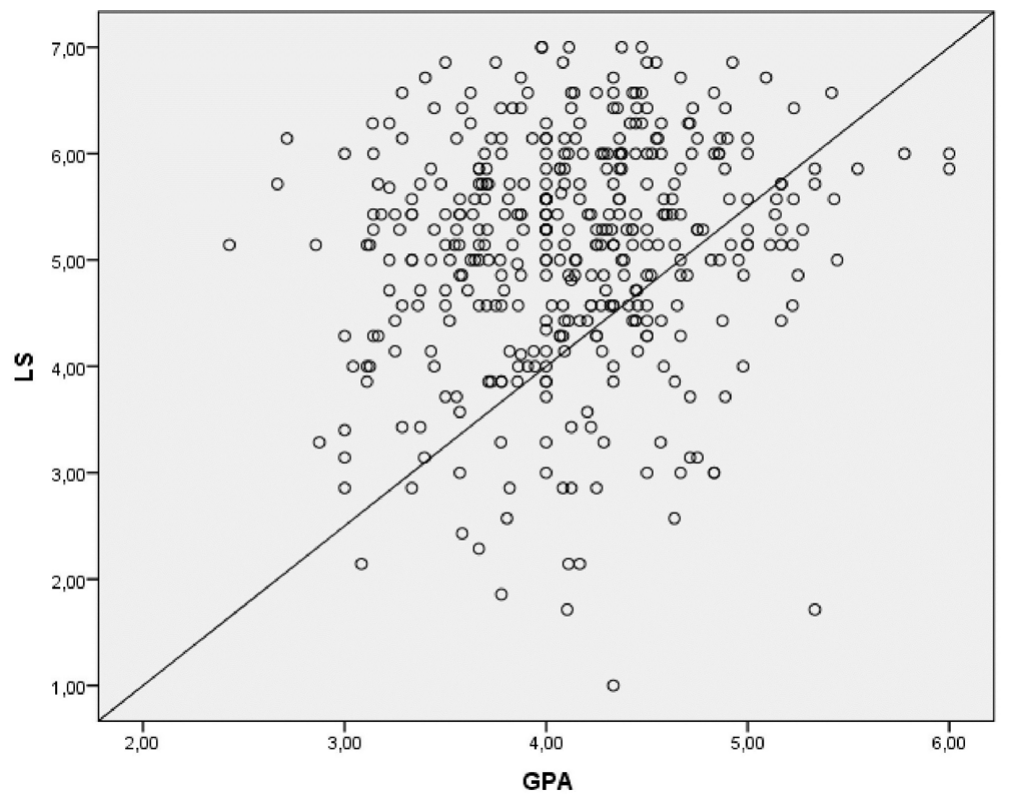
**Relationship between academic achievement [grade point average (GPA)] and life satisfaction (LS), *r* = 0.14**.

With regard to the first hypothesis, students’ academic achievement (GPA) and LS scores were weakly but significantly positively correlated (*r* = 0.14). **Figure 1** displays a scatter plot of students’ indicators of academic achievement and LS.

### MODERATOR ANALYSES

Our second hypothesis that parents’ education moderated the association between students’ LS and academic achievement was only partly confirmed (see **Table 3**).

**Table 3:** Regression of LS on students’ grades, examining the moderating effect of mothers’ education (Model 1) and fathers’ education (Model 2).

	*Beta*	*T*	*P*	*R^2^*
**Model 1**
Academic achievement	0.40	3.70	<0.01	
Mothers’ education	0.13	-1.16	<0.05	
Academic achievement × mothers’ education	0.47	-2.42	<0.05	
				0.04
**Model 2**
Academic achievement	0.29	2.38	<0.05	
Fathers’ education	-0.03	-0.32	<0.05	
Academic achievement × fathers’ education	-0.05	-0.29	>0.05	
				0.02

**FIGURE 2:**
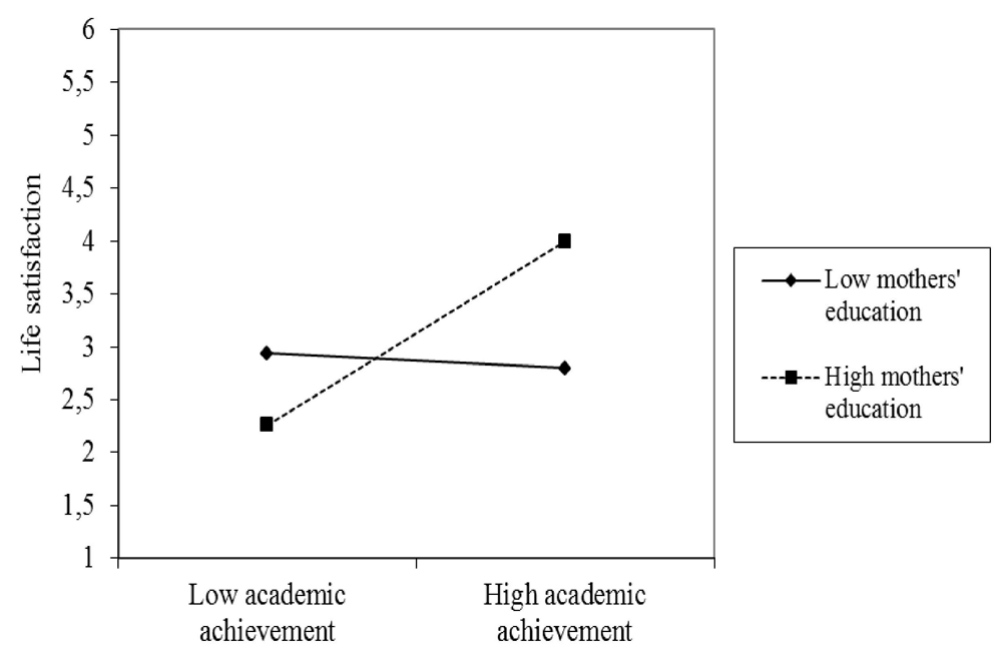
**Mothers’ education as a moderator of students’ LS and academic achievement**.

Whilst fathers’ education did not moderate the association between students’ LS scores and academic achievement (*t* = -0.29, *p* > 0.05), mothers’ education did (*t* = -2.42, *p* < 0.05). We further explored in what way the association was different for students with a low and high education background from their mother. Simple slope analyses showed that academic achievement and LS were significantly positively correlated for students who attended the same school track as their parents (β = 0.23, *t* = 3.70, *p* < 0.01), but not for students who attended a higher school track than their parents (β = -0.04, *t* = 0.44, *p* = 0.66). Thus, academic achievement was only positively associated with LS for students attending the same school track as their parents did but not for students attending a higher school track than their parents did. This is illustrated in **Figure 2**.

In sum, mothers’ education had a moderating role for the association between children’s LS and academic achievement, but fathers’ education did not.

## DISCUSSION

The aim of the present paper was to explore the interplay between adolescents’ academic achievement and LS and the moderating role of parents’ education in a sample of German high school students. The results contribute to the discussion of adolescents’ LS in the context of education which has only gained particular attention recently (Suldo et al., 2006; Ferguson et al., 2011), potentially as a response to academically rigorous school environments in today’s competitive society. As studies investigating adolescents’ LS in relation to education are still scarce (Suldo et al., 2006), our study may shed light on the background mechanisms of the heterogeneous relationship between academic achievement and students’ LS found in earlier studies.

As we hypothesized, students’ academic achievement was positively correlated to LS, which has been reported in other studies before (e.g., Verkuyten and Thijs, 2002). However, in the light of results found in other studies with similar sample sizes, our results hinge on a comparably lower correlation. A reason for the lower correlation in the present study might be that our sample is highly selective as it consists of students attending a Gymnasium, the highest school track in Germany in which students from disadvantaged social backgrounds and/or migration backgrounds are mostly underrepresented. Moreover, we used GPA (grades obtained by students’ report cards) as a measure of academic achievement whereas other studies have assessed academic achievement by *self-reported* measures of grades (Gilman and Huebner, 2006) or standardized assessment measures (Suldo and Shaffer, 2008). For self-reported grades it has been found that their validity is different from those of objectively obtained grades (Kuncel et al., 2005) which might explain the differences between the studies. Furthermore, the correlation found in the sample of students who attended a school track as high as the one their parents attended in the current study was comparable with that of the other studies. It might be that the other studies primarily investigated student samples that followed their parents’ academic track. However, this information is not stated in the previous studies. Whether the differences between our results and those of other studies are due to sample characteristics or methodological aspects should be examined in further studies.

We further examined whether parents’ education moderates the relationship between students’ academic achievement and LS, hypothesizing that for students who attend a higher school track than their parents, the association is lower than for students who attend a school track equal to their parents. First, we demonstrated that mothers’ education indeed functioned as a moderator of the association between academic achievement and LS. The association between academic achievement and LS was limited to those students whose mothers have the same or higher education than their children. Surprisingly, this moderating function found for mothers’ education was not found for fathers’ education. We expected parents’ education to be a moderator of the given association in general but did not specifically expect any differential results for mothers’ and fathers’ education. Yet our results are in line with findings demonstrating that mothers’ education alone predicts children’s academic success (Klebanov et al., 1994; Davis-Kean, 2005).

It is already known that parents from high academic educational backgrounds demonstrate higher educational aspirations for their children (Zhan and Sherraden, 2003; Kim and Sherraden, 2011), yet the question still remains why mothers may play a more significant role than fathers concerning their children’s LS in the context of education. A possible explanation for this result may lie in the fact that German mothers, even if they are highly educated and have a job, often do more parenting work than fathers (e.g., Allmendinger, 2009) and consequently are more often confronted with their children’s school issues and general life concerns. Especially in Western Germany, the number of mothers returning to work after childbirth is much smaller than in East Germany and most of the European countries (Bredtmann et al., 2009). Moreover, many women and mothers work part-time in Western Germany (Kreyenfeld, 2000) and German policies are known to facilitate traditional gender roles, i.e., women predominantly acting as mothers and housewives, while men are seen as “breadwinners” (Trappe and Rosenfeld, 2000). As parental education and parent-child communication about school have been found to be positively related to children’s positive academic outcomes (Grolnick and Slowiaczek, 1994), it is plausible that parenting work, including communication about education, is mostly done by mothers in Western Germany and consequently affects children’s school issues and satisfaction with life more than fathers do. Thus, it might be that mothers’ interactions with their children are more frequent than those of fathers and eventually more important for those aspects of children’s subjective well-being that are related to their daily lives, such as school issues.

However, there was no association between academic performance and LS for students who attended a higher school track than their mothers. This can be explained by the idea that children in these families are already seen as academically successful by outperforming their parents in a higher school track and are consequently less confronted with high educational expectations by their parents. Furthermore, as suggested by theories of social inequalities and educational attainment, parents from low socio-economic backgrounds are less powerful in participating in questions of education affecting their children’s academic success (Bourdieu and Passeron, 1971), especially if their children follow an academic track unfamiliar to them. Thus, they are less likely to get involved in their children’s school-related activities and are thus less prone to put pressure and expectations on their children. It might be that well-educated mothers put more pressure on their children to succeed in school to ensure that their children keep up the social status the parents have attained (Schnabel et al., 2002).

However, these ideas are only possible explanations for our results and future studies in this field should focus more on assessing aspects of parental involvement. As many studies within educational research put a great emphasis on analyzing academic pressures and examination stress in schools (e.g., Rana and Mahmood, 2010) and the negative effects of academically rigorous environments (e.g., Marsh and Hau, 2003), it would be interesting to examine whether parents and their interactions with their children may be an even greater source of academic pressure for children from higher socio-economic backgrounds.

## LIMITATIONS

The cross-sectional nature of the present study means that it cannot be assured which reasons account for the differential effects of mothers’ and fathers’ education on the relation between students’ academic achievement and LS. For greater details, the analyses undertaken in the present study should be replicated with longitudinal data to clarify the direction of the effects. Furthermore, without more information about parents’ background, especially about their career paths as well as other variables such as gender role-distribution, it is not possible to fully explain these differences. Consequently, further studies should examine more parental background information.

Moreover, the investigated sample consisted of students who attended a Gymnasium, the highest and most selective school track in Germany. Therefore, our results cannot be transferred to other types of schools. However, it would still be interesting to examine how the relationship between academic success and LS turns out to be in other types of schools if students attend a school type lower than the one their parents attended.

## CONCLUSION AND FUTURE DIRECTION

Finally, the present study tried to give insight into factors influencing the relationship between academic achievement and LS. So far, research on children’s LS has predominantly examined intra-personal variables (for a review see Huebner, 2004) and there has been a lack of studies considering contextual factors as potential moderators of the respective association.

Our results underline that education research must continue to assess students’ SES to help understanding the effects of family not only on academic performance (Sirin, 2005), but also on students’ well-being. Moreover, the present study also provides an indication that highly educated mothers are a key resource in Western Germany, impacting their children’s academic performance and LS whereas in today’s society fathers appear to have less impact on these aspects of their children’s life. Therefore, the results of this study may give reason to critically engage in questions about gender roles and family in modern society of Western Germany today.

Another interesting direction through which to extend this investigation is to examine differences concerning gender and migration background of high and low SES students. Well-educated mothers with migration background have been found to have a strong impact on children’s academic achievement as they are more likely in favor of supporting education with results that are more in favor of girls than for boys (Ara, 2012; Nielsen and Rangvid, 2012). Therefore, the impact of mother’s education found in the present study might be further moderated by gender and/or students’ migration background, which could be tested using larger samples. Eventually, this direction could provide further insight into understanding the underlying mechanisms of the impact of mothers’ education on children’s school success and LS.

## Conflict of Interest Statement

The authors declare that the research was conducted in the absence of any commercial or financial relationships that could be construed as a potential conflict of interest.
